# The Effect of Intravascular Imaging-Guided Percutaneous Coronary Intervention on Coronary Artery Perforation

**DOI:** 10.1016/j.jacasi.2024.09.004

**Published:** 2024-11-12

**Authors:** Yuichi Sawayama, Kenta Sasaki, Narumi Taninobu, Akihiro Ikuta, Kohei Osakada, Shunsuke Kubo, Takeshi Tada, Yasushi Fuku, Hiroyuki Tanaka, Yoshihisa Nakagawa, Kazushige Kadota

**Affiliations:** a Department of Cardiovascular Medicine, Kurashiki Central Hospital, Kurashiki, Japan; b Department of Cardiovascular Medicine, Shiga University of Medical Science, Otsu, Japan

**Keywords:** coronary artery perforation, intravascular imaging, percutaneous coronary intervention

## Abstract

**Background:**

Intravascular imaging (IVI) complements coronary angiography and may help prevent coronary artery perforation (CAP) during percutaneous coronary intervention (PCI).

**Objectives:**

The authors evaluated whether IVI-guided PCI is associated with a lower risk of PCI-related CAP in a real-world cohort.

**Methods:**

This observational study analyzed consecutive PCI procedures from January 2006 to October 2023. The procedures were divided into 4 groups according to the year performed: 2006 to 2010 (P1), 2011 to 2015 (P2), 2016 to 2020 (P3), and 2021 to 2023 (P4). We evaluated the trend of IVI-guided PCI and the incidence of CAP. A mixed effects logistic model was employed to assess their relationship.

**Results:**

CAP occurred in 368 (1.6%) of 22,368 PCIs. IVI-guided PCI accounted for 63% of all cases, of which 95% were intravascular ultrasound procedures. From P1 to P3, the ratio of IVI-guided PCI increased linearly (P1: 30%, P2: 61%, P3: 93%, P4: 97%), while the incidence of CAP decreased (P1: 2.10%, P2: 1.74%, P3: 1.13%, P4: 1.18%). IVI-guided PCI showed a significant association with a lower risk of the overall incidence of CAP (adjusted OR: 0.78; 95% CI: 0.61-0.99; *P =* 0.047). This relationship was particularly significant for chronic total occlusion PCI (adjusted OR: 0.59; 95% CI: 0.43-0.80; *P =* 0.001) and PCI for moderate or severe calcification (adjusted OR: 0.50; 95% CI: 0.33-0.74; *P =* 0.001).

**Conclusions:**

IVI-guided PCI may help prevent PCI-related CAP, especially in the setting of chronic total occlusion PCI and PCI for moderate or severe calcification.

Coronary artery perforation (CAP) during percutaneous coronary intervention (PCI) is a rare yet potentially severe complication in current clinical practice. The reported incidence rate of CAP during PCI procedures ranges from 0.1% to 2.0% overall,^1^ with a trend of increasing incidence over time.^2^ Notably, this rate is higher at approximately 4% for chronic total occlusion (CTO) procedures,^3^ and it increases to 15% when using the retrograde approach.^4^ CAP is associated with adverse cardiac events during hospital stay, including cardiac tamponade, hemodynamic instability, and death.^5^ Therefore, operators should take a proactive approach to minimize the development of PCI-related CAP.

Intravascular imaging (IVI), including intravascular ultrasound (IVUS) and optical coherence tomography (OCT), complements coronary angiography in terms of assessing vessels and guiding lesion preparation, stent deployment, and optimal endpoints.^6^^,^^7^ Two randomized controlled trials recently showed that IVI-guided PCI led to a lower risk of cardiovascular events after PCI than did angiography-guided PCI in patients with complex coronary artery lesions.^8^^,^^9^ IVI also has utility for the assessment of PCI-related complications. Poststenting IVI can detect stent malapposition and stent edge dissection, which may increase the risk of stent thrombosis, myocardial infarction, or repeat revascularization.^10^ Extramural hematomas can also be confirmed by IVI.^6^^,^^7^ Additionally, IVI before PCI can provide lesion information, and achieving an appropriate balloon or stent size theoretically lowers the risk of CAP. However, this is inconsistent with existing data on IVI-guided PCI itself being a risk factor for CAP.^11^^,^^12^ Notably, in these previous studies, IVI was not routinely used in practice, instead being favored for more complex lesions. This introduces the possibility of bias that could have affected the study outcomes.

IVI is now routinely used in Japan in part because of reimbursement for PCI, with IVI-guided PCI accounting for more than 80% of all cases.^13^ In this study, we evaluated whether IVI-guided PCI is associated with a lower risk of CAP in a real-world cohort. We also examined this association by vessel type of CAP.

## Methods

### Data source

This observational study analyzed consecutive PCI procedures performed at Kurashiki Central Hospital from January 2006 to October 2023. The PCI procedures were included regardless of clinical presentation or procedure details. Detailed data were obtained from the medical records and angiographic images. The study was performed in accordance with the principles of the Declaration of Helsinki. Because of the study’s retrospective nature, the local medical ethics committee waived the requirement for informed consent. Instead, an opt-out procedure was implemented.

### Percutaneous coronary imaging

PCI was performed using a 6- to 8-F guiding catheter. Unfractionated heparin was administered (100 U/kg), and the activated clotting time was monitored every 30 to 60 minutes. The operator then titrated the heparin to maintain the activated clotting time at ≥250 seconds. Debulking devices were used as necessary. The decision regarding the utilization of IVI, and if used, the selection between IVUS or OCT, was left to the operator’s discretion. The primary objectives of IVI were to achieve comprehensive coverage of the coronary artery lesion with a stent, ensure optimal stent expansion, and minimize instances of stent malapposition. During the OCT procedure, blood was removed by flushing with low-molecular-weight dextran or contrast medium through a guide catheter. Although the timing of IVI use was at the discretion of the operators, it was used predilation, postpreparation, and poststenting in most cases. Cases in which IVI was used only after CAP to evaluate or manage extravasation were treated as non–IVI-guided PCI in the data analyses.

### Classification and management of CAP

Perforated vessels were categorized as main, distal, or collateral.^14^ Main vessel perforation was classified using the Ellis system (types I to IV).^15^ CAP management was at the operator’s discretion. Pericardiocentesis or mechanical circulatory support was implemented as needed in patients with hemodynamic instability.

### Definitions

Renal impairment was defined as current renal replacement therapy or an estimated glomerular filtration rate of <45 mL/min/1.73 m^2^.^16^ Moderate or severe calcification was defined as a radiopaque density visible at least during the cardiac cycle before contrast injection.^17^ PCI for bifurcation lesions was defined as a procedure that implemented kissing balloon inflation, regardless of stenting in the main or side branches.

### Statistical analysis

Summary statistics for demographic and clinical characteristics are provided for the study cohort and the subgroups of patients who did and did not develop CAP. Continuous variables with a normal distribution are presented as mean ± SD and were compared using the Student’s *t*-test, whereas those with a skewed distribution are presented as median (IQR) and were compared using the Wilcoxon rank-sum test. Categorical variables are presented as number with proportion and were compared using the chi-square test. The PCI procedures were divided into 4 groups according to the year performed: 2006 to 2010 (P1), 2011 to 2015 (P2), 2016 to 2020 (P3), and 2021 to 2023 (P4). The Cochran-Armitage test for categorical variables was used to assess trends among the groups.

To determine the association of IVI-guided PCI with CAP occurrence, mixed effects logistic regression was used to calculate adjusted ORs and 95% CIs. Covariates as fixed effects were determined based on clinical knowledge from prior studies^2^^,^^11^^,^^12^ and the availability of data, including age (≥75 years or not), sex, renal impairment (yes or no), indication for PCI (acute or chronic coronary syndrome), PCI for CTO (yes or no), PCI for moderate or severe calcification (yes or no), PCI for a bifurcation lesion (yes or no), rotational atherectomy use (yes or no), and IVI use (yes or no). We treated the year category when PCI procedures were performed (P1 to P4) as random effects. We also employed the model by CAP type (ie, CAP in main, distal, or collateral vessels). In addition, we performed subgroup analyses with interaction tests in the clinically relevant subgroups, including age 75 years or older, prior PCI, PCI for CTO, PCI for moderate or severe calcification, and PCI with rotational atherectomy. The mixed effects logistic regression model was the same as the main analysis, but an indication for PCI was not included in the model for subgroup analysis of CTO PCI because PCI for acute coronary syndrome (ACS) and CTO rarely coexist.

Furthermore, we calculated 30-day and 5-year mortality in patients with and without CAP. To minimize the effect of short-term mortality, we conducted a landmark analysis beyond 30 days. In patients who underwent more than one PCI within the study period, the initial PCI was analyzed. For patients with CAP, the first PCI in which CAP developed was analyzed, even if the patient had undergone a prior PCI within the study period. The cumulative incidence of 30-day and 5-year mortality was estimated using the Kaplan-Meier method. We also calculated adjusted HRs and 95% CIs with a Cox proportional hazard regression model. We included the following variables in the multivariable model: age, sex, diabetes mellitus (yes or no), renal impairment (estimated glomerular filtration rate of <45 mL/min/1.73 m^2^ or not), and indication for PCI (acute or chronic syndrome).^2^ All reported *P* values were 2-sided, and a *P* value <0.05 was considered statistically significant. Statistical analyses were performed using SAS software version 9.4 (SAS Institute) and JMP version 16 (SAS Institute).

## Results

### Patient and procedure characteristics

CAP occurred in 368 (1.6%) of 22,368 PCIs during the study period. The patients’ baseline clinical characteristics are presented in Table 1. Their mean age was 70.3 ± 11.2 years, and 17,071 (76%) were men. PCI for ACS, including ST-segment elevation myocardial infarction and non–ST-segment elevation ACS, was included in 7,386 (33%), and PCI for CTO was in 2,884 (13%). IVI was used in 14,007 (63%), of which 13,297 (95%) were IVUS.

**Table 1:** Baseline Clinical Characteristics According to Presence or Absence of CAP

	Overall (N = 22,368)	CAP (n = 368)	No CAP (n = 22,000)	*P* Value
Patient characteristics				
Age, y	70.3 ± 11.2	72.4 ± 10.4	70.3 ± 11.2	<0.001
Male	17,071 (76)	277 (75)	16,794 (76)	0.634
Body mass index, kg/m^2^	24.0 ± 3.7	23.2 ± 3.6	24.0 ± 3.7	<0.001
Current smoking	1,388 (6)	47 (13)	1,341 (6)	<0.001
Hypertension	17,752 (79)	257 (70)	17,495 (80)	<0.001
Diabetes mellitus	9,838 (44)	125 (34)	9,713 (44)	<0.001
Dyslipidemia	15,782 (71)	231 (63)	15,551 (71)	0.001
Renal impairment	4,880 (22)	91 (25)	4,789 (22)	<0.001
Peripheral vascular disease	1,269 (6)	49 (13)	1,220 (6)	<0.001
Prior myocardial infarction	8,270 (37)	199 (54)	8,071 (37)	<0.001
Prior cerebral infarction	1,992 (9)	28 (8)	1,964 (9)	0.379
Prior PCI	10,759 (48)	216 (59)	10,543 (48)	<0.001
Prior CABG	1,019 (5)	32 (9)	987 (4)	<0.001
Procedure characteristics				
Clinical presentation				<0.001
STEMI	3,807 (17)	27 (7)	3,780 (17)	
NSTE-ACS	3,579 (16)	30 (8)	3,549 (16)	
CCS	14,982 (67)	311 (85)	14,671 (67)	
No. of vessels with disease				0.016
1 vessel	13,333 (60)	197 (54)	13,136 (60)	
2 vessels	4,870 (22)	80 (22)	4,790 (22)	
3 vessels	2,080 (9)	50 (14)	2,030 (9)	
LMCA	192 (0.9)	2 (0.5)	190 (0.9)	
LMCA and 1 vessel	649 (3)	10 (3)	639 (3)	
LMCA and 2 vessels	773 (3)	21 (6)	752 (3)	
LMCA and 3 vessels	471 (2)	8 (2)	463 (2)	
PCI for CTO	2,884 (13)	179 (49)	2,705 (12)	<0.001
PCI for moderate or severe calcification	3,938 (18)	127 (35)	3,811 (17)	<0.001
PCI for bifurcation lesion	7,293 (33)	112 (30)	7,181 (33)	0.371
PCI with rotational atherectomy	1,206 (5)	50 (14)	1,156 (5)	<0.001
IVI-guided PCI	14,007 (63)	224 (61)	13,783 (63)	0.484
IVUS use	13,297 (59)	221 (60)	13,076 (59)	0.811
OCT use	710 (3)	3 (0.8)	707 (3)	0.009

Values are mean ± SD or n (%). *P* values were calculated between patients with and without CAP.

CABG = coronary artery bypass grafting; CAP = coronary artery perforation; CCS = chronic coronary syndrome; CTO = chronic total occlusion; IVI = intravascular imaging; IVUS = intravascular ultrasound; LMCA = left main coronary artery; NSTE-ACS = non–ST-segment elevation acute coronary syndrome(s); OCT = optical coherence tomography; PCI = percutaneous coronary intervention; STEMI = ST-segment elevation myocardial infarction.

Relative to patients without CAP, patients with CAP were older, were more often current smokers, and more frequently had a history of myocardial infarction, PCI, and coronary artery bypass grafting. ACS as the clinical presentation was less prevalent in the CAP group (57 of 368 [15%]) than in the no CAP group (7,329 of 22,000 [33%]) (*P <* 0.001). PCI for CTO was more frequently performed in patients with than without CAP (CAP 179 [49%] vs no CAP 2,705 [12%]; *P* < 0.001). The ratio of IVI-guided PCI procedures was similar between the 2 groups (CAP 224 [61%] vs no CAP 13,783 [63%]; *P =* 0.484). In the setting of CTO PCI, the retrograde approach and J-CTO score ≥2 were more frequently observed in the CAP group (Supplemental Table 1).

Periprocedural adverse events in patients with and without CAP were available in Supplemental Table 2. Patients with CAP showed a slightly higher rate of periprocedural myocardial infarction and cardiovascular death within 30 days, but neither was substantially significant.

### Classification, management, and outcomes of CAP

The characteristics of CAP, including its classification, site of perforation, and management, are shown in Table 2. Among a total of 368 CAP cases, the main, distal, and collateral vessel perforations were in 165 (45%), 160 (43%), and 43 (12%), respectively. Of the main vessel perforations, the Ellis classification was type I in 22 (13%), type II in 81 (49%), type III in 56 (34%), and type IV in 6 (4%). The most common site of perforation was the right coronary artery in 162 (44%), followed by the left circumflex artery in 83 (23%) and left anterior descending artery in 65 (18%). CAP classification and perforated site were similar for the IVI- and angiography-guided PCI group.

**Table 2:** Classification and Management of CAP

	Overall (N = 368)	IVI-Guided PCI (n = 224)	Angiography-Guided PCI (n = 144)	*P* Value
CAP classification				0.675
Ellis I	22 (6.0)	14 (6.3)	8 (5.6)	
Ellis II	81 (22.0)	48 (21.4)	33 (22.9)	
Ellis III	56 (15.2)	31 (13.8)	25 (17.4)	
Ellis IV	6 (1.6)	5 (2.2)	1 (0.7)	
Distal vessel	160 (43.5)	102 (45.5)	58 (40.3)	
Collateral vessel	43 (11.7)	24 (10.7)	19 (13.2)	
Perforated site				0.590
LMCA	2 (0.5)	2 (0.9)	0 (0)	
LAD	65 (17.7)	44 (19.6)	21 (14.6)	
LCX	83 (22.6)	53 (23.7)	30 (20.8)	
RCA	162 (44.0)	92 (41.1)	70 (48.6)	
Diagonal branch	24 (6.5)	15 (6.7)	9 (6.3)	
High lateral branch	6 (1.6)	4 (1.8)	2 (1.4)	
Septal branch	26 (7.1)	14 (6.3)	12 (8.3)	
Hemostatic procedures				
Balloon tamponade	269 (73.1)	173 (77.2)	96 (66.7)	0.026
Perfusion balloon	14 (3.8)	14 (6.3)	0 (0)	0.002
Covered stent	27 (7.3)	18 (8.0)	9 (6.3)	0.521
Stent^a^	34 (9.2)	21 (9.4)	13 (9.0)	0.911
Coil	57 (15.5)	36 (16.1)	21 (14.6)	0.700
Fat embolization	29 (7.9)	15 (6.7)	14 (9.7)	0.293

Values are n (%).

LAD = left anterior descending artery; LCX = left circumflex artery; RCA = right coronary artery; other abbreviations as in Table 1.

a Used only for bailout.

Management and in-hospital outcomes in patients with CAP are available in Supplemental Table 3. Overall, pericardiocentesis was implemented in 16 (4.4%) and in 8 (12.9%) of 62 with Ellis type III or IV perforations. CAP-related death occurred in 4 patients: 2 with main vessel perforations (both Ellis type III or IV) and 2 with distal vessel perforations. Once CAP had occurred, whether IVI guided the PCI or not did not substantially affect the outcomes.

### Temporal trend of PCI and CAP

**Figure 1:**
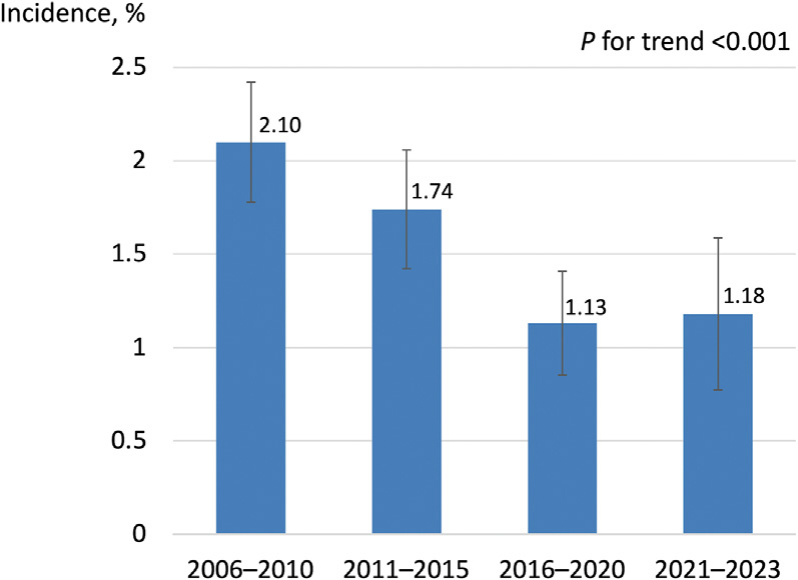
Temporal Trend of Coronary Artery Perforation Incidence The incidence of coronary artery perforation (CAP) decreased from P1 to P3 and plateaued thereafter. Error bars indicated 95% CIs.

The temporal trend of PCI procedures and CAP incidence is shown in Table 3. Throughout the study period, the number of PCI procedures performed per year decreased over time, but the number of patients with ACS remained relatively constant at approximately 400 per year. PCI for chronic coronary syndrome and CTO decreased with time. There was an increasing trend in PCI for moderate or severe calcification, while the utilization of rotational atherectomy remained generally unchanged across the study period. The ratio of IVI-guided PCI linearly increased from 30% in P1 to 93% in P3, with a near-plateau thereafter (*P* for trend < 0.001). The incidence of CAP decreased from P1 to P3 and plateaued thereafter (P1: 2.10% [95% CI: 1.78-2.42], P2: 1.74% [95% CI: 1.42-2.06], P3: 1.13% [95% CI: 0.85-1.41], P4: 1.18% [95% CI: 0.77-1.59]; *P* for trend < 0.001) (Figure 1). This decreasing trend of CAP was consistently observed regardless of CAP type (Central Illustration). Trends in guidewire types contributing to CAP in distal vessels showed a decline in the use of polymer-jacketed wires over time, with coil wires being used in 80% of cases of PCI-related CAP in recent years (Supplemental Table 4).

**Table 3:** Temporal Trend of PCI Procedures and Incidence of CAP

	2006-2010 (P1) (N = 7,627)	2011-2015 (P2) (N = 6,478)	2016-2020 (P3) (N = 5,559)	2021-2023 (P4) (N = 2,704)	*P* Value for Trend
PCI procedures					
PCI for ACS	2,108 (28)	2,053 (32)	2,050 (37)	1,175 (43)	<0.001
PCI for CTO	1,268 (17)	820 (13)	551 (10)	245 (9)	<0.001
PCI for moderate or severe calcification	1,049 (14)	1,048 (16)	1,197 (22)	644 (24)	<0.001
PCI for bifurcation lesion	2,327 (31)	2,203 (34)	1,929 (35)	834 (31)	0.012
PCI with rotational atherectomy	464 (6.1)	314 (4.9)	264 (4.8)	164 (6.1)	0.133
IVI-guided PCI	2,283 (30)	3,938 (61)	5,163 (93)	2,623 (97)	<0.001
CAP incidence					
Main vessel perforation	72 (0.94)	55 (0.85)	23 (0.41)	15 (0.55)	<0.001
Distal vessel perforation	64 (0.84)	46 (0.71)	35 (0.63)	15 (0.55)	0.076
Collateral vessel perforation	24 (0.31)	12 (0.19)	5 (0.09)	2 (0.07)	0.001
Main vessel perforation in CTO PCI	38 (3.0)	22 (2.7)	9 (1.6)	10 (4.1)	0.747
Distal vessel perforation in CTO PCI	28 (2.2)	14 (1.7)	12 (2.2)	2 (1.2)	0.453
Collateral vessel perforation in CTO PCI	24 (1.9)	12 (1.5)	5 (0.9)	2 (0.8)	0.069

Values are n (%)

ACS = acute coronary syndrome(s); other abbreviations as in Table 1.

**Central Illustration:**
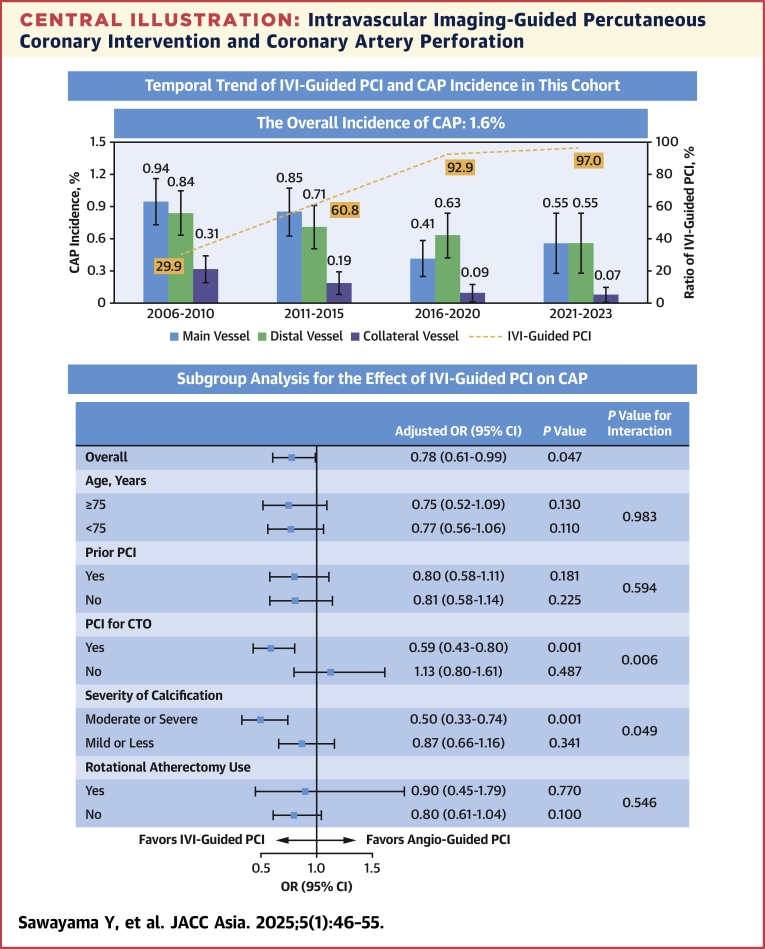
Intravascular Imaging-Guided Percutaneous Coronary Intervention and Coronary Artery Perforation (Top) Throughout the study period, the ratio of intravascular imaging (IVI)-guided percutaneous coronary intervention (PCI) increased over time, with an inverse decrease in the incidence of coronary artery perforation (CAP). Error bars indicated 95% CIs. (Bottom) Subgroup analysis showed that IVI-guided PCI was significantly associated with a lower risk of CAP in the setting of chronic total occlusion (CTO) PCI and PCI for moderate or severe calcification.

### Association between IVI-guided PCI and CAP

Table 4 summarizes the predictors of PCI-related CAP. IVI-guided PCI showed a significant association with a lower risk of the overall incidence of CAP (adjusted OR: 0.78; 95% CI: 0.61-0.99; *P =* 0.047). PCI for CTO (adjusted OR: 5.95, 95% CI: 4.72-7.50; *P* < 0.001) and PCI for moderate or severe calcification (adjusted OR: 1.92; 95% CI: 1.49-2.47; *P* < 0.001) were robustly associated with an increased risk of CAP. The results of the analyses by CAP type are shown in Supplemental Table 5. A substantial association between IVI-guided PCI and CAP incidence was not found in each CAP type (main vessel: adjusted OR: 0.70; 95% CI: 0.49-1.00; *P =* 0.051; distal vessels: adjusted OR: 0.87; 95% CI: 0.62-1.21; *P =* 0.395; collateral vessel: adjusted OR: 1.11; 95% CI: 0.56-2.21; *P =* 0.760). For Ellis type III or IV CAP, the result was similar (adjusted OR: 0.61; 95% CI: 0.36-1.04; *P =* 0.067) (Supplemental Table 6).

**Table 4:** Multivariable Analysis for Predictors of CAP

	Unadjusted		Adjusted	
	OR (95% CI)	*P* Value	OR (95% CI)	*P* Value
Age ≥75 y	1.29 (1.05-1.58)	0.017	1.38 (1.11-1.72)	0.004
Male	0.94 (0.74-1.20)	0.634	0.95 (0.74-1.22)	0.678
Renal impairment	1.18 (0.93-1.50)	0.173	1.04 (0.81-1.33)	0.765
Prior PCI	1.54 (1.25-1.90)	<0.001	1.28 (1.03-1.60)	0.026
PCI for ACS	0.37 (0.28-0.49)	<0.001	0.81 (0.59-1.13)	0.214
PCI for CTO	6.76 (5.49-8.32)	<0.001	5.95 (4.72-7.50)	<0.001
PCI for moderate or severe calcification	2.52 (2.02-3.13)	<0.001	1.92 (1.49-2.47)	<0.001
PCI for bifurcation lesion	0.90 (0.72-1.13)	0.371	0.94 (0.75-1.18)	0.583
PCI with rotational atherectomy	2.84 (2.09-3.84)	<0.001	1.41 (0.99-2.01)	0.054
IVI-guided PCI	0.93 (0.75-1.15)	0.484	0.78 (0.61-0.99)	0.047

Abbreviations as in Tables 1 and 3.

A subgroup analysis was shown in the Central Illustration. The effect of IVI-guided PCI on the overall incidence of CAP was generally consistent across subgroups. Particularly, a significant association was observed in the setting of CTO PCI (adjusted OR: 0.59; 95% CI: 0.43-0.80; *P =* 0.001, *P* for interaction = 0.006) and PCI for moderate or severe calcification (adjusted OR: 0.50; 95% CI: 0.33-0.74; *P =* 0.001, *P* for interaction = 0.049). These results were primarily driven by the lower risk of CAP in main vessels (Supplemental Table 7).

### Short- and long-term mortality in patients with and without CAP

**Figure 2:**
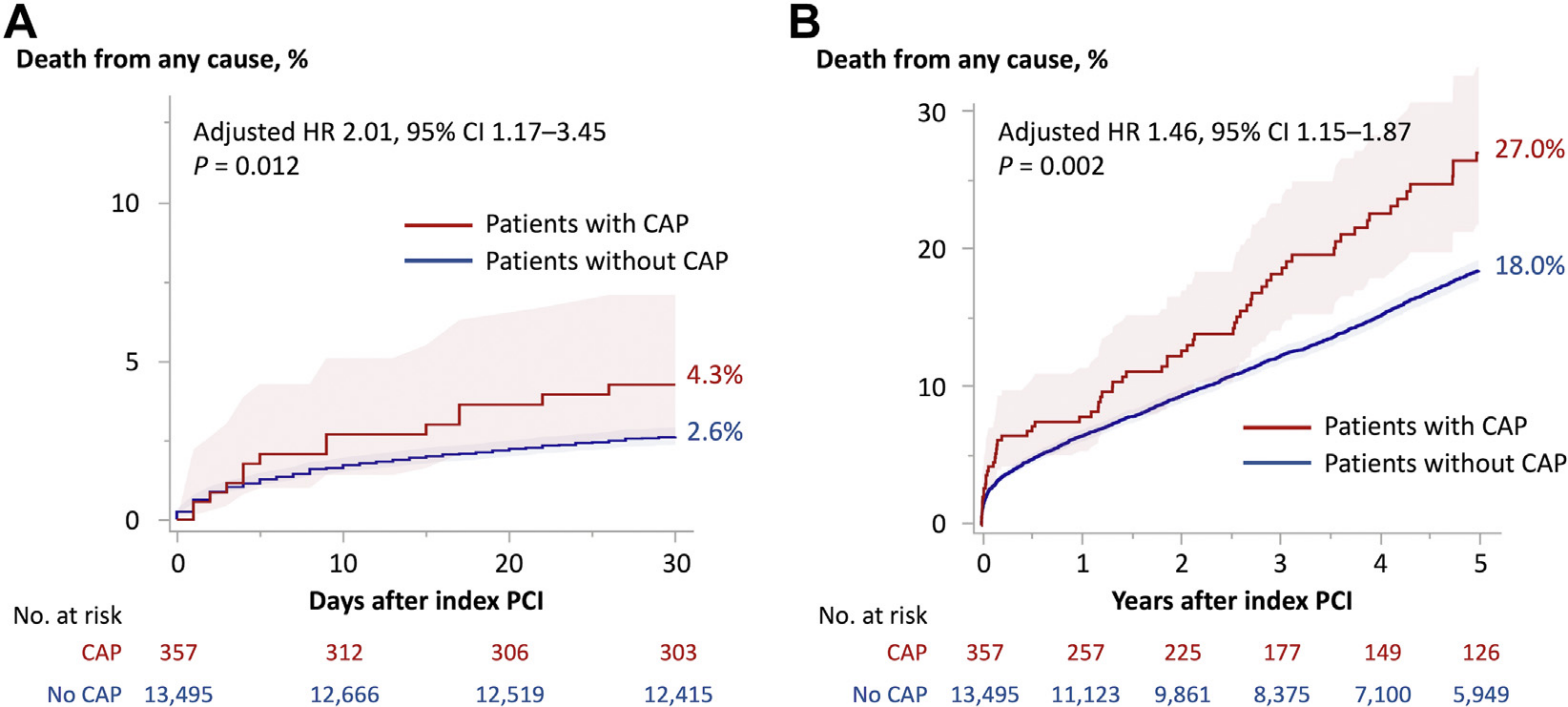
Kaplan-Meier Curves of Death From Any Cause Patients with coronary artery perforation (CAP) (red line) vs those without CAP (blue line) over a duration of (A) 30 days and (B) 5 years. Shaded regions indicated 95% CIs. PCI = percutaneous coronary intervention.

The median follow-up duration was 1,090 days (Q1-Q3: 246-2,526 days) in patients who developed CAP (n = 357) and 1,557 days (Q1-Q3: 632-3,102 days) in those who did not (n = 13,495). The cumulative 30-day mortality rate for patients with CAP was 4.3%, which was significantly higher than the rate of 2.6% in patients without CAP (adjusted HR: 2.01; 95% CI: 1.17-3.45; *P =* 0.012) (Figure 2A). The cumulative 5-year mortality rate was still significantly higher in patients with than without CAP (27.0% vs 18.0%, respectively; adjusted HR: 1.46; 95% CI: 1.15-1.87; *P =* 0.002) (Figure 2B). This result was mostly consistent with the landmark analysis beyond 30 days (adjusted HR: 1.38; 95% CI: 1.05-1.81; *P =* 0.022) (Supplemental Figure 1).

## Discussion

In the current study, we analyzed 22,368 PCIs (IVI-guided PCI: 14,007, angiography-guided PCI: 8,361) in a real-world cohort of all-comer patients during an approximately 18-year period. The overall incidence of CAP was fairly high at 1.6%. Throughout the study period, the ratio of IVI-guided PCI increased over time, with an inverse decrease in the incidence of CAP. This decreasing trend of CAP was consistently observed regardless of CAP type. Our study showed that IVI-guided PCI was significantly associated with a lower risk of overall CAP and, importantly, may be particularly effective in preventing CAP in some situations (CTO PCI and PCI for moderate or severe calcification) (Central Illustration). In addition, patients who developed CAP had a higher mortality rate in both the short- and long-term periods than patients who did not develop CAP.

We found that IVI-guided PCI was significantly associated with a lower risk of CAP, especially in the setting of CTO PCI and PCI for moderate or severe calcification. This result seems plausible from a theoretical point of view but inconsistent with previous reports.^11^^,^^12^ The most likely conceivable explanation is that IVI was not routinely used in previous studies. As stated within one of the previously mentioned reports,^12^ IVI was more likely to be used in complex lesions or complicated situations such as balloon underexpansion during predilation. This implies a possibility of bias that led to the strong association with IVI-guided PCI and CAP occurrence. In our cohort, however, IVI-guided PCI was more routinely performed. Especially since 2016, the rate of IVI-guided PCI has indeed exceeded 90%. Another possible reason may be that some patients required IVI after the onset of CAP to elucidate the mechanism underlying persistent extravasation or to ensure appropriate management of extravasation. Indeed, in 22 cases in our cohort, IVI was used only after the onset of CAP (we treated these cases as non–IVI-guided PCI). Dealing with these cases as IVI-guided PCI would have led to a stronger correlation between IVI-guided PCI and CAP onset.

Employing IVI before intervention can facilitate evaluation of vessel diameter, plaque composition, and localization of calcification, enabling selection of the optimal balloon size and stent diameter.^6^ These effects may collectively contribute to the prevention of CAP in main vessels. In our analysis, CTO PCI presented a notable risk factor for CAP. The higher risk of perforation in more complex CTO lesions (ie, J-CTO score ≥2) aligns with the previous studies.^18^ The underlying mechanism may involve perforation caused by the CTO wire or the specific nature of the treatment via the collateral channel. In the former scenario, the IVI before balloon dilation can mitigate the risk of serious perforation. In addition, our cohort exhibited a significant association between CAP and PCI performed on lesions with moderate or severe calcification. The calcified nodule^19^ or the C-type calcified and residual thin plaque sign (C-CAT sign)^20^ is recognized as a contributory factor for CAP in main vessels. For heavily calcified lesions, it is crucial to identify whether these findings exist using IVI. Furthermore, guidewire bias is a factor that can predict coronary artery dissection or perforation in PCI with rotational atherectomy, which has particularly important implications in cases involving tortuous or angulated vessels. Given that implementing rotational atherectomy has a potential risk factor for CAP, identifying guidewire bias using IVI is crucial. In contrast, theoretically, IVI does not seem to be beneficial in preventing distal vessel perforation. Indeed, our results showed no potential effectiveness of IVI-guided PCI in preventing CAP in distal vessels, but its incidence also indicated a decreasing trend. The underlying contributory factors might be the decrease in CTO PCI, along with a decrease in the use of stiff wires and the lower frequency of using polymer-jacketed wires.

As in previous studies,^2^^,^^21^^,^^22^ patients who developed CAP in our cohort exhibited higher short- and long-term mortality rates than patients who did not, even after adjusting for covariates. In addition, a continued excess mortality risk after 30 days was observed, which is consistent with the findings of a prior study.^22^ Generally, the age at which PCI is performed is increasing each year, and there is a concomitant increase in the complexity of PCI.^23^ Given the potential link between CAP and mortality risk in both the early and late phases, operators are encouraged to minimize the risk of CAP occurrence during PCI. The results of the current study highlight the potential benefits of incorporating IVI routine use in clinical practice for achieving this goal.

### Study limitations

First, as a nonrandomized retrospective observational study, this study was inherently susceptible to certain biases. In addition to selection bias, existing data have other limitations including the imprecision inherent in clinical data and protocol variation in CAP management. In our cohort, balloon tamponade accounted for only 73% of CAP treatments, and whether the CAP treatment algorithm^24^ was followed may have affected the clinical outcomes. Furthermore, the number of variables was limited, and measured or unmeasured confounders were present. Although we constructed a mixed effects model and adjusted possible confounding factors, this limitation cannot be completely eliminated. Second, the treatment strategy based on IVI was not strictly standardized and was at the discretion of the operators. This may have affected the study outcomes. Third, 95% of IVI devices in our cohort were IVUS devices, and it might be difficult to extend our results to OCT-guided PCI. However, OCT imaging offers significantly enhanced visualization of the interface between the lumen and plaque surface, allowing for more precise lumen measurement than capable with IVUS.^25^ Fourth, whether CAP is directly or indirectly associated with death from any cause was not elucidated in this study. However, the higher mortality risk in patients who developed CAP, as well as the fact that its effect extended to the later phase after CAP onset, are consistent with the findings of prior studies.^2^^,^^21^^,^^22^

## Conclusions

This study showed that the ratio of IVI-guided PCI increased and that inversely, the incidence of CAP decreased in a real-world setting during an approximately 18-year period. IVI-guided PCI was associated with a lower risk of CAP, especially in main vessels, in the setting of CTO PCI and PCI for moderate or severe calcification. This suggests that routine implementation of IVI-guided PCI may be beneficial in preventing PCI-related CAP in these situations.

## Funding Support and Author Disclosures

The authors have reported that they have no relationships relevant to the contents of this paper to disclose.

## References

[1] Mikhail P., Howden N., Monjur M. (2022). Coronary perforation incidence, outcomes and temporal trends (COPIT): a systematic review and meta-analysis. Open Heart.

[2] Ford T.J., Adamson C., Morrow A.J. (2022). Coronary artery perforations: Glasgow Natural History Study of Covered Stent Coronary Interventions (GNOCCI) Study. J Am Heart Assoc.

[3] Danek B.A., Karatasakis A., Tajti P. (2017). Incidence, treatment, and outcomes of coronary perforation during chronic total occlusion percutaneous coronary intervention. Am J Cardiol.

[4] Wu K., Huang Z., Zhong Z. (2019). Predictors, treatment, and long-term outcomes of coronary perforation during retrograde percutaneous coronary intervention via epicardial collaterals for recanalization of chronic coronary total occlusion. Catheter Cardiovasc Interv.

[5] Lemmert M.E., Van Bommel R.J., Diletti R. (2017). Clinical characteristics and management of coronary artery perforations: a single-center 11-year experience and practical overview. J Am Heart Assoc.

[6] Truesdell A.G., Alasnag M.A., Kaul P. (2023). Intravascular imaging during percutaneous coronary intervention: JACC state-of-the-art review. J Am Coll Cardiol.

[7] Saito Y., Kobayashi Y., Fujii K. (2024). CVIT 2023 clinical expert consensus document on intravascular ultrasound. Cardiovasc Interv Ther.

[8] Lee J.M., Choi K.H., Song Y.B. (2023). Intravascular imaging–guided or angiography-guided complex PCI. N Engl J Med.

[9] Holm N.R., Andreasen L.N., Neghabat O. (2023). OCT or angiography guidance for PCI in complex bifurcation lesions. N Engl J Med.

[10] Räber L., Mintz G.S., Koskinas K.C. (2018). Clinical use of intracoronary imaging. Part 1: guidance and optimization of coronary interventions. An expert consensus document of the European Association of Percutaneous Cardiovascular Interventions. Eur Heart J.

[11] Bauer T., Boeder N., Nef H.M. (2015). Fate of patients with coronary perforation complicating percutaneous coronary intervention (from the Euro Heart Survey Percutaneous Coronary Intervention Registry). Am J Cardiol.

[12] Al-Lamee R., Ielasi A., Latib A. (2011). Incidence, predictors, management, immediate and long-term outcomes following grade III coronary perforation. JACC Cardiovasc Interv.

[13] Kuno T., Miyamoto Y., Numasawa Y. (2024). Enhancing coronary intervention outcomes using intravascular ultrasound: analysis of long-term benefits in a Japanese multicenter registry. J Soc Cardiovasc Angiogr Interv.

[14] Muller O., Windecker S., Cuisset T. (2008). Management of 2 major complications in the cardiac catheterisation laboratory: the no-reflow phenomenon and coronary perforations. EuroIntervention.

[15] Ellis S.G., Ajluni S., Arnold A.Z. (1994). Increased coronary perforation in the new device era. Incidence, classification, management, and outcome. Circulation.

[16] Arora S., Stouffer G.A., Kucharska-Newton A. (2018). Fifteen-year trends in management and outcomes of non-st-segment-elevation myocardial infarction among Black and White patients: the ARIC community surveillance study, 2000-2014. J Am Heart Assoc.

[17] Kim M.C., Ahn Y., Sim D.S. (2017). Impact of calcified bifurcation lesions in patients undergoing percutaneous coronary intervention using drug-eluting stents: results from the COronary BIfurcation Stent (COBIS) II registry. EuroIntervention.

[18] Kostantinis S., Simsek B., Karacsonyi J. (2022). Incidence, mechanisms, treatment, and outcomes of coronary artery perforation during chronic total occlusion percutaneous coronary intervention. Am J Cardiol.

[19] Morofuji T., Kuramitsu S., Shinozaki T. (2021). Clinical impact of calcified nodule in patients with heavily calcified lesions requiring rotational atherectomy. Catheter Cardiovasc Interv.

[20] Kawamura A., Egami Y., Nishino M., Tanouchi J. (2023). The C-CAT sign may predict coronary artery perforation in severe calcified lesions during coronary intervention: a case series. Eur Heart J Case Rep.

[21] Parsh J., Seth M., Green J. (2017). Coronary artery perforations after contemporary percutaneous coronary interventions: evaluation of incidence, risk factors, outcomes, and predictors of mortality. Catheter Cardiovasc Interv.

[22] Kinnaird T., Anderson R., Ossei-Gerning N. (2017). Legacy effect of coronary perforation complicating percutaneous coronary intervention for chronic total occlusive disease. Circ Cardiovasc Interv.

[23] Protty M., Sharp A.S.P., Gallagher S. (2022). Defining percutaneous coronary intervention complexity and risk: an analysis of the United Kingdom BCIS Database 2006-2016. JACC Cardiovasc Interv.

[24] Doll J.A., Hira R.S., Kearney K.E. (2020). Management of percutaneous coronary intervention complications: algorithms from the 2018 and 2019 Seattle Percutaneous Coronary Intervention Complications Conference. Circ Cardiovasc Interv.

[25] Kubo T., Akasaka T., Shite J. (2013). OCT compared with IVUS in a coronary lesion assessment: the OPUS-CLASS study. JACC Cardiovasc Imaging.

