# Supplementary material for: The Effect of Intravascular Imaging-Guided Percutaneous Coronary Intervention on Coronary Artery Perforation
Source: JACC Asia. 2024 Nov 12;5(1):46–55. doi: 10.1016/j.jacasi.2024.09.004 (PMC11775771; doi:10.1016/j.jacasi.2024.09.004)
Supplement: Supplemental Tables 1-7 and Supplemental Figure 1 [file mmc1.docx]

**The Effect of Intravascular Imaging-Guided Percutaneous Coronary Intervention on Coronary Artery Perforation**

**Running title:** Intravascular Imaging and CAP

Yuichi Sawayama, MD^a,b^; Kenta Sasaki, MD^a^; Narumi Taninobu, MD^a^; Akihiro Ikuta, MD^a^; Kohei Osakada, MD^a^; Shunsuke Kubo, MD^a^; Takeshi Tada, MD^a^; Yasushi Fuku, MD^a^; Hiroyuki Tanaka, MD^a^; Yoshihisa Nakagawa, MD^b^; Kazushige Kadota, MD^a^

**SUPPLEMENTAL MATERIALS**

Authors: Sawayama et al.

**Supplemental Table 1.** CAP in CTO PCI

**Supplemental Table 2.** Periprocedural adverse events in patients with and without CAP

**Supplemental Table 3.** Management and In-Hospital Outcomes in Patients with CAP

**Supplemental Table 4.** Guidewire Types Causing CAP in Distal Vessels

**Supplemental Table 5.** Multivariable Analyses for Predictors of CAP by Type

**Supplemental Table 6.** Multivariable Analyses for Predictors of Ellis Type III or IV CAP

**Supplemental Table 7.** Subgroup Analysis for the Effect of IVI-Guided PCI on CAP by Type

**Supplemental Figure 1.** Kaplan–Meier Curves for Death from Any Cause in Landmark Analysis Beyond 30 Days

**Supplemental Table 1.** CAP in CTO PCI

|  | Overall  (N = 2,884) | CAP  (N = 179) | No CAP  (N = 2,705) | *P* value |
| --- | --- | --- | --- | --- |
| IVI-guided PCI | 2,004 (69.5) | 103 (57.5) | 1,901 (70.3) | <0.001 |
| PCI for moderate or severe calcification | 697 (24.2) | 59 (33.0) | 638 (23.6) | 0.005 |
| PCI with rotational atherectomy | 253 (8.8) | 17 (9.5) | 236 (8.7) | 0.723 |
| Guidewire approach |  |  |  | <0.001 |
| Antegrade | 2,109 (73.1) | 89 (49.7) | 2,020 (74.7) |  |
| Retrograde | 775 (26.9) | 90 (50.3) | 685 (25.3) |  |
| J-CTO score |  |  |  | <0.001 |
| ≥2 | 636 (22.1) | 77 (43.0) | 559 (20.7) |  |
| <2 | 2,248 (77.9) | 102 (57.0) | 2,146 (79.3) |  |

Data are presented as n (%). *P* values were calculated between patients with and without CAP.

CAP, coronary artery perforation; CTO, chronic total occlusion; IVI, intravascular imaging; PCI, percutaneous coronary intervention.

**Supplemental Table 2.** Periprocedural adverse events in patients with and without CAP

|  | Overall | CAP | No CAP | *P* value |
| --- | --- | --- | --- | --- |
| Periprocedural MI (CK-MB >5× UNL) | 1,139/14,982 (7.6) | 28/311 (9.0) | 1,111/14,671 (7.6) | 0.346 |
| Periprocedural MI (CK-MB >10× UNL) | 692/14,982 (4.6) | 15/311 (4.8) | 677/14,671 (4.6) | 0.862 |
| Target vessel MI within 30 days | 49/22,368 (0.2) | 0/368 (0) | 49/22,000 (0.2) | 0.368 |
| Cardiovascular death within 30 days | 356/13,852 (2.6) | 12/357 (3.4) | 344/13,495 (2.6) | 0.338 |
| MI | 277/356 (78) | 5/12 (42) | 272/344 (79) |  |
| Cardiac tamponade | 4/356 (1.1) | 4/12 (33) | 0/344 (0) |  |
| Heart failure | 54/356 (15) | 2/12 (17) | 52/344 (15) |  |
| Ventricular arrhythmia | 11/356 (3.1) | 0/12 (0) | 11/344 (3.2) |  |
| Aortic disease | 4/356 (1.1) | 0/12 (0) | 4/344 (1.2) |  |
| Stroke | 6/356 (1.7) | 1/12 (8.3) | 5/344 (1.5) |  |

Data are presented as n (%). For periprocedural MI, only PCIs for chronic coronary syndrome were included. For cardiovascular death within 30 days, consecutive patients were analyzed.

CAP, coronary artery perforation; CK-MB, creatine kinase-MB; MI, myocardial infarction; UNL, upper limit of normal

**Supplemental Table 3.** Management and In-Hospital Outcomes in Patients with CAP

|  | Overall | Main vessel | Distal vessel | Collateral vessel | Ellis type III or IV |
| --- | --- | --- | --- | --- | --- |
| **Overall** | **N = 368** | **N = 165** | **N = 160** | **N = 43** | **N = 62** |
| Ischemic stroke | 9 (2.5) | 6 (3.6) | 3 (1.9) | 0 (0) | 4 (6.5) |
| Pericardiocentesis | 16 (4.4) | 10 (6.1) | 4 (2.5) | 2 (4.7) | 8 (12.9) |
| More than one intervention for hemostasis | 4 (1.1) | 2 (1.2) | 2 (1.3) | 0 (0) | 0 (0) |
| Cardiac surgery | 7 (1.9) | 4 (2.4) | 3 (1.9) | 0 (0) | 4 (6.5) |
| CAP-related death | 4 (1.1) | 2 (1.2) | 2 (1.3) | 0 (0) | 2 (3.2) |
| Death from any cause | 18 (4.9) | 7 (4.2) | 11 (6.9) | 0 (0) | 3 (4.8) |
| **IVI-guided PCI** | **N = 224** | **N = 100** | **N = 102** | **N = 22** | **N = 39** |
| Ischemic stroke | 4 (1.8) | 3 (3.0) | 1 (1.0) | 0 (0) | 2 (5.1) |
| Pericardiocentesis | 12 (5.4) | 8 (8.2) | 3 (2.9) | 1 (4.6) | 6 (16.7) |
| More than one intervention for hemostasis | 4 (1.8) | 2 (2.0) | 2 (2.0) | 0 (0) | 0 (0) |
| Cardiac surgery | 6 (2.7) | 4 (4.0) | 2 (2.0) | 0 (0) | 4 (10.3) |
| CAP-related death | 3 (1.3) | 2 (2.0) | 1 (1.0) | 0 (0) | 2 (5.1) |
| Death from any cause | 8 (3.6) | 2 (2.0) | 6 (5.9) | 0 (0) | 2 (5.1) |
| **Angiography-guided PCI** | **N = 144** | **N = 65** | **N = 58** | **N = 21** | **N = 23** |
| Ischemic stroke | 5 (3.5) | 3 (4.6) | 2 (3.5) | 0 (0) | 2 (8.7) |
| Pericardiocentesis | 4 (2.8) | 2 (3.0) | 1 (1.7) | 1 (4.8) | 2 (8.7) |
| More than one intervention for hemostasis | 0 (0) | 0 (0) | 0 (0) | 0 (0) | 0 (0) |
| Cardiac surgery | 1 (0.7) | 0 (0) | 1 (1.7) | 0 (0) | 0 (0) |
| CAP-related death | 1 (0.7) | 0 (0) | 1 (1.7) | 0 (0) | 0 (0) |
| Death from any cause | 10 (6.9) | 5 (7.7) | 5 (8.6) | 0 (0) | 1 (4.4) |

Data are presented as n (%). Ischemic stroke was defined as a new neurological deficit in the context of an acute cerebral infarction visualized on magnetic resonance imaging.

CAP, coronary artery perforation.

**Supplemental Table 4.** Guidewire Types Causing CAP in Distal Vessels

|  | 2006–2010 (P1) | 2011–2015 (P2) | 2016–2020 (P3) | 2021–2023 (P4) |
| --- | --- | --- | --- | --- |
| Coil wire | 23 (36) | 30 (65) | 25 (71) | 12 (80) |
| Polymer-jacketed wire | 38 (59) | 14 (30) | 6 (17) | 2 (13) |
| Stiff wire | 3 (4.7) | 2 (4.4) | 4 (11.4) | 1 (6.7) |

Data are presented as n (%).

CAP, coronary artery perforation.

**Supplemental Table 5.** Multivariable Analyses for Predictors of CAP by Type

|  | Main vessel | | Distal vessel | | Collateral vessel | |
| --- | --- | --- | --- | --- | --- | --- |
|  | Adjusted OR (95% CI) | *P* value | Adjusted OR (95% CI) | *P* value | Adjusted OR (95% CI) | *P* value |
| Age ≥75 years | 1.07 (0.77–1.48) | 0.688 | 1.88 (1.36–2.60) | <0.001 | 0.85 (0.45–1.64) | 0.632 |
| Male | 1.10 (0.75–1.60) | 0.629 | 1.00 (0.69–1.46) | 0.998 | 0.60 (0.31–1.17) | 0.133 |
| Renal impairment | 1.62 (1.15–2.26) | 0.005 | 0.76 (0.51–1.14) | 0.185 | 0.21 (0.07–0.69) | 0.010 |
| Prior PCI | 1.29 (0.93–1.80) | 0.121 | 1.26 (0.91–1.75) | 0.169 | 1.70 (0.90–3.20) | 0.100 |
| PCI for ACS | 0.68 (0.41–1.13) | 0.141 | 0.90 (0.58–1.38) | 0.619 | — | — |
| PCI for CTO | 4.97 (3.55–6.96) | <0.001 | 3.66 (2.57–5.23) | <0.001 | — | — |
| PCI for moderate or severe calcification | 3.13 (2.20–4.47) | <0.001 | 1.00 (0.64–1.54) | 0.983 | 3.07 (1.52–6.20) | 0.002 |
| PCI for bifurcation lesion | 0.64 (0.44–0.92) | 0.018 | 1.78 (1.30–2.44) | <0.001 | 0.10 (0.02–0.41) | 0.002 |
| PCI with rotational atherectomy | 1.35 (0.85–2.14) | 0.208 | 2.00 (1.13–3.54) | 0.018 | 0.43 (0.10–1.91) | 0.264 |
| IVI-guided PCI | 0.70 (0.49–1.00) | 0.051 | 0.87 (0.62–1.21) | 0.395 | 1.11 (0.56–2.21) | 0.760 |

For CAP in main and distal vessels, adjustments were made for age, sex, renal impairment (yes or no), indication for PCI (acute or chronic coronary syndrome), PCI for CTO (yes or no), PCI for moderate or severe calcification (yes or no), PCI for bifurcation lesion (yes or no), rotational atherectomy use (yes or no), and IVI use (yes or no). For CAP in collateral vessels, PCI for ACS and PCI for CTO were not included in the multivariable model because all patients underwent PCI for CTO.

The year category was treated as random effects.

ACS, acute coronary syndrome; CAP, coronary artery perforation; CI, confidence interval; CTO, chronic total occlusion; IVI, intravascular imaging; OR, odds ratio; PCI, percutaneous coronary intervention.

**Supplemental Table 6.** Multivariable Analyses for Predictors of Ellis Type III or IV CAP

|  | Ellis type III or IV | |
| --- | --- | --- |
|  | Adjusted OR (95% CI) | *P* value |
| Age ≥75 years | 1.20 (0.71–2.02) | 0.496 |
| Male | 1.14 (0.62–2.08) | 0.674 |
| Renal impairment | 1.35 (0.78–2.33) | 0.282 |
| Prior PCI | 1.31 (0.77–2.22) | 0.323 |
| PCI for ACS | 0.89 (0.42–1.86) | 0.756 |
| PCI for CTO | 3.69 (2.12–6.43) | <0.001 |
| PCI for moderate or severe calcification | 2.67 (1.45–4.91) | 0.002 |
| PCI for bifurcation lesion | 0.37 (0.18–0.76) | 0.006 |
| PCI with rotational atherectomy | 2.94 (1.49–5.78) | 0.002 |
| IVI-guided PCI | 0.61 (0.36–1.04) | 0.067 |

Adjustments were made for age, sex, renal impairment (yes or no), indication for PCI (acute or chronic coronary syndrome), PCI for CTO (yes or no), PCI for moderate or severe calcification (yes or no), PCI for bifurcation lesion (yes or no), rotational atherectomy use (yes or no), and IVI use (yes or no).

The year category was treated as random effects.

ACS, acute coronary syndrome; CAP, coronary artery perforation; CI, confidence interval; CTO, chronic total occlusion; IVI, intravascular imaging; OR, odds ratio; PCI, percutaneous coronary intervention.

|  | Main vessel | | | Distal vessel | | | Collateral vessel | | |
| --- | --- | --- | --- | --- | --- | --- | --- | --- | --- |
|  | Adjusted OR (95% CI) | *P* value | *P* value for interaction | Adjusted OR (95% CI) | *P* value | *P* value for interaction | Adjusted OR (95% CI) | *P* value | *P* value for interaction |
| Age, years |  |  | 0.291 |  |  | 0.730 |  |  | 0.043 |
| ≥75 | 0.54 (0.31–0.94) | 0.029 |  | 0.84 (0.53–1.34) | 0.470 |  | 3.59 (0.92–14.0) | 0.066 |  |
| <75 | 0.81 (0.52–1.27) | 0.362 |  | 0.89 (0.55–1.43) | 0.628 |  | 1.06(0.53–2.13) | 0.876 |  |
| Prior PCI |  |  | 0.352 |  |  | 0.607 |  |  | — |
| Yes | 0.63 (0.39–0.99) | 0.047 |  | 0.93 (0.59–1.47) | 0.754 |  | 1.01 (0.44–2.35) | 0.975 |  |
| No | 0.85 (0.51–1.43) | 0.548 |  | 0.81 (0.49–1.32) | 0.396 |  | — | — |  |
| PCI for CTO* |  |  | 0.099 |  |  | 0.144 |  |  | — |
| Yes | 0.55 (0.34–0.88) | 0.013 |  | 0.66 (0.38–1.15) | 0.142 |  | — | — |  |
| No | 1.03 (0.62–1.72) | 0.901 |  | 1.04 (0.68–1.57) | 0.862 |  | — | — |  |
| Severity of calcification |  |  | 0.058 |  |  | 0.400 |  |  | — |
| Moderate or severe | 0.40 (0.24–0.69) | 0.001 |  | 1.21 (0.51–2.82) | 0.667 |  | 0.20 (0.06–0.60) | 0.005 |  |
| Mild or less | 0.89 (0.57–1.38) | 0.593 |  | 0.81 (0.56–1.17) | 0.258 |  |  |  |  |
| PCI with rotational atherectomy |  |  | 0.515 |  |  | 0.141 |  |  | — |
| Yes | 0.44 (0.18–1.08) | 0.072 |  | 2.81 (0.62–12.8) | 0.179 |  | — | — |  |
| No | 0.74 (0.51–1.09) | 0.131 |  | 0.79 (0.56–1.12) | 0.185 |  | 1.06 (0.53–2.13) | 0.876 |  |

**Supplemental Table 7.** Subgroup Analysis for the Effect of IVI-Guided PCI on CAP by Type

For CAP in main and distal vessels, adjustments were made for age (≥75 years or not), sex, renal impairment (yes or no), indication for PCI (acute or chronic coronary syndrome), PCI for CTO (yes or no), PCI for moderate or severe calcification (yes or no), PCI for bifurcation lesion (yes or no), rotational atherectomy use (yes or no), and IVI use (yes or no). For CAP in collateral vessels, PCI for ACS and PCI for CTO were not included in the multivariable model because all patients underwent PCI for CTO. In addition, among patients who developed CAP in collateral vessels, there were few patients without prior PCI, those without moderate of severe calcification, and those with rotational atherectomy; therefore, the corresponding OR and 95% CI were not calculated.

*PCI for ACS was not included in the multivariable model.

ACS, acute coronary syndrome; CAP, coronary artery perforation; CI, confidence interval; CTO, chronic total occlusion; IVI, intravascular imaging; OR, odds ratio; PCI, percutaneous coronary intervention.

**Supplemental Figure 1.** Kaplan–Meier Curves for Death from Any Cause in Landmark Analysis Beyond 30 Days


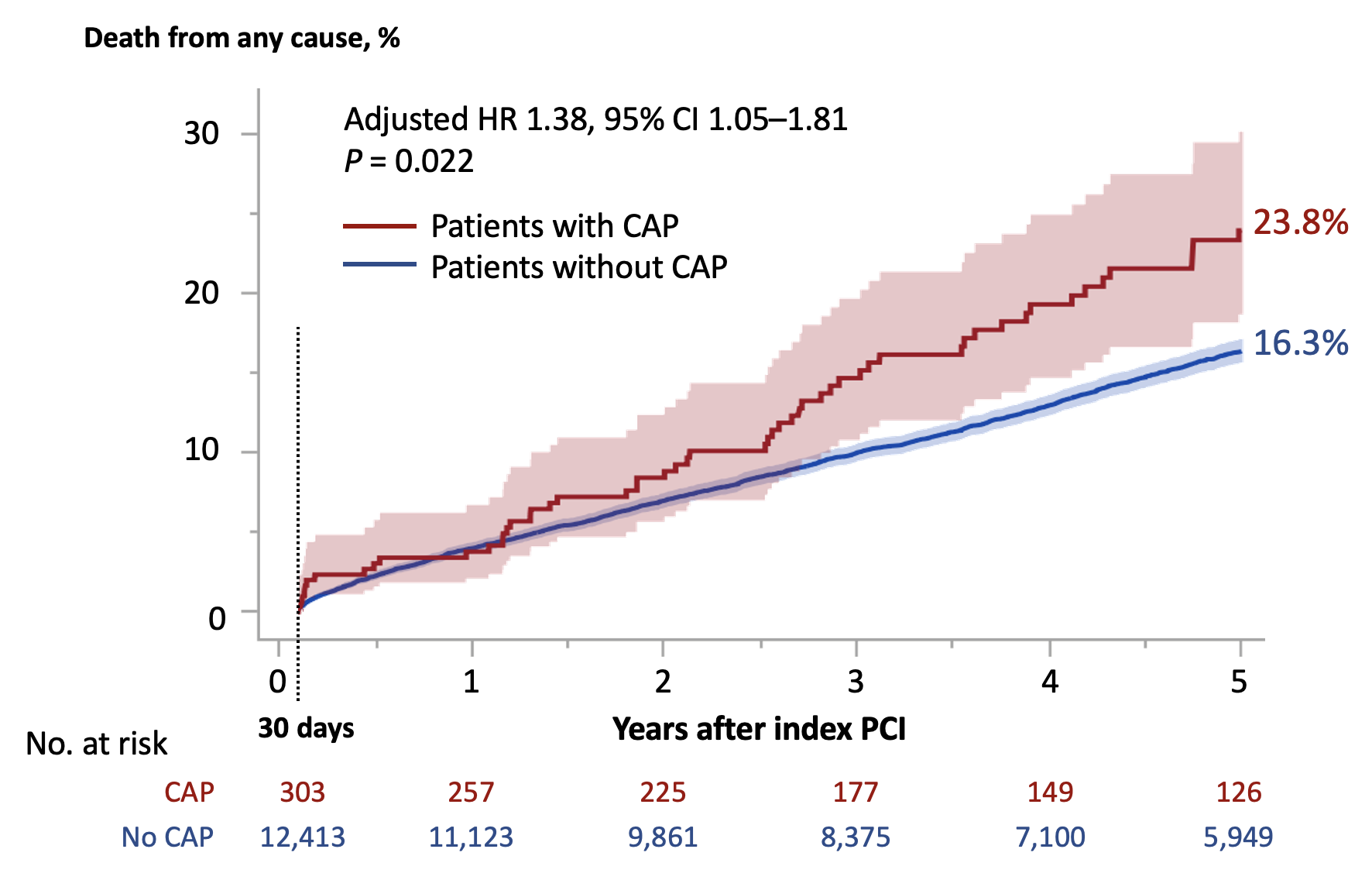
Patients who died or missed follow-up within 30 days after the index PCI were excluded from the landmark analysis beyond 30 days. Hazard ratios (HRs) and 95% confidence intervals (CIs) were calculated by the Cox proportional hazard regression model. The following variables were included in the multivariable model: age, sex, diabetes mellitus (yes or no), renal impairment (estimated glomerular filtration rate of <45 mL/min/1.73 m^2^ or not), and indication for PCI (acute or chronic syndrome)
